# Crystal structure, Hirshfeld surface analysis and HOMO–LUMO analysis of (*E*)-*N*′-(3-hy­droxy-4-meth­oxy­benzyl­idene)nicotinohydrazide monohydrate

**DOI:** 10.1107/S2056989019006492

**Published:** 2019-05-14

**Authors:** Palaniyappan Sivajeyanthi, Bellarmin Edison, Kasthuri Balasubramani, Ganesan Premkumar, Toka Swu

**Affiliations:** a Department of Chemistry, Government Arts College (Autonomous), Thanthonimalai, Karur 639 005, Tamil Nadu, India; b Department of Chemistry, Pondicherry University, R.V. Nagar, Kalapet, Puducherry 605 014, India

**Keywords:** crystal structure, Schiff base, inter­molecular inter­actions, Hirshfeld surface analysis

## Abstract

The mol­ecule of the title Schiff base compound, C_14_H_13_N_3_O_3_·H_2_O, displays a *trans* configuration with respect to the C=N bond. The dihedral angle between the benzene and pyridine rings is 29.63 (7)°. The crystal structure features inter­molecular N—H⋯O, C—H⋯O, O—H⋯O and O—H⋯N hydrogen-bonding inter­actions, leading to the formation of a supramolecular framework. A Hirshfeld surface analysis indicates that the most important contributions to the crystal packing are from H⋯H (37.0%), O⋯H/H⋯O (23.7%)), C⋯H/H⋯C (17.6%) and N⋯H/H⋯N (11.9%) inter­actions. The title compound has also been characterized by frontier mol­ecular orbital analysis.

## Abstract 2

The title Schiff base compound displays an *E* configuration with respect to the C=N double bond. The pyridine and benzene rings subtend a dihedral angle of 29.63 (7)°. In the crystal, the mol­ecules are linked by N—H⋯O, C—H⋯O, O—H⋯O and O—H⋯N hydrogen-bonding inter­actions.

## Chemical context   

Schiff bases are nitro­gen-containing compounds that were first obtained by the condensation reaction of aromatic amines and aldehydes (Schiff, 1864 ▸). A wide range of these compounds, with the general formula *R*HC=N*R*_1_ (*R* and *R*_1_ can be alkyl, aryl, cyclo­alkyl or heterocyclic groups) have been synthesized. Schiff bases are of great importance in the field of coordination chemistry because they are able to form stable complexes with metal ions (Souza *et al.*, 1985 ▸). The chemical and biological significance of Schiff bases can be attributed to the presence of a lone electron pair in the *sp* ^2^-hybridized orbital of the nitro­gen atom of the azomethine group (Singh *et al.*, 1975 ▸). These compounds are used in the fields of organic synthesis, chemical catalysis, medicine and pharmacy, as well as other new technologies (Tanaka *et al.*, 2010 ▸). Schiff bases are also used as probes for investigating the structure of DNA (Tiwari *et al.*, 2011 ▸) and have gained special attention in pharmacophore research and in the development of several bioactive lead mol­ecules (Muralisankar *et al.*, 2016 ▸). Schiff bases showing photochromic and thermochromic properties have been used in information storage, electronic display systems, optical switching devices and ophthalmic glasses (Amimoto *et al.*, 2005 ▸). As a further contribution to this field of research, we report herein the crystal structure of the title compound, (*E*)-*N*′-(3-hy­droxy-4-meth­oxy­benzyl­idene)nicotinohydrazide monohydrate.
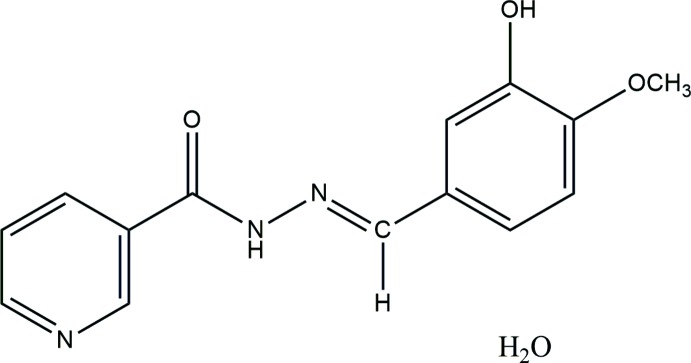


## Structural commentary   

**Figure 1:**
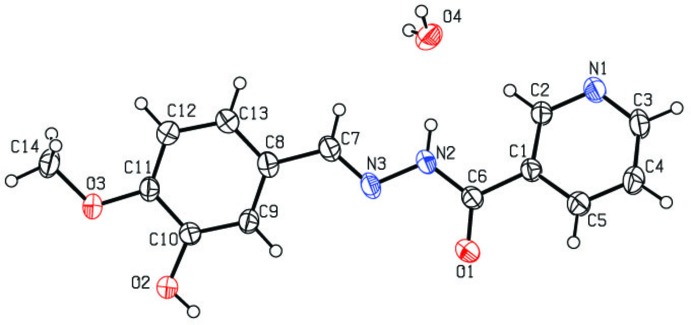
The asymmetric unit of the title compound with displacement ellipsoids drawn at the 50% probability level..

The asymmetric unit of the title compound (Fig. 1 ▸) consists of one independent Schiff base mol­ecule displaying a *trans* configuration with respect to the C=N bond and a water mol­ecule. All the bond lengths are within the normal ranges (Allen *et al.*, 1987 ▸). The C7=N3 bond length of 1.274 (2) Å is consistent with a double-bond character. The C6—N2 and N2—N3 bond lengths of 1.343 (2) and 1.3866 (16) Å, respectively, are comparable to those observed in related compounds (Sivajeyanthi *et al.*, 2017 ▸; Balasubramani *et al.*, 2018 ▸). The O1/C6/N2/N3/C7 core is almost planar (r.m.s. deviation = 0.022 Å) and forms dihedral angles of 20.75 (7) and 8.93 (5)°, respectively, with the pyridine and benzene rings.

## Supra­molecular features   

**Figure 2:**
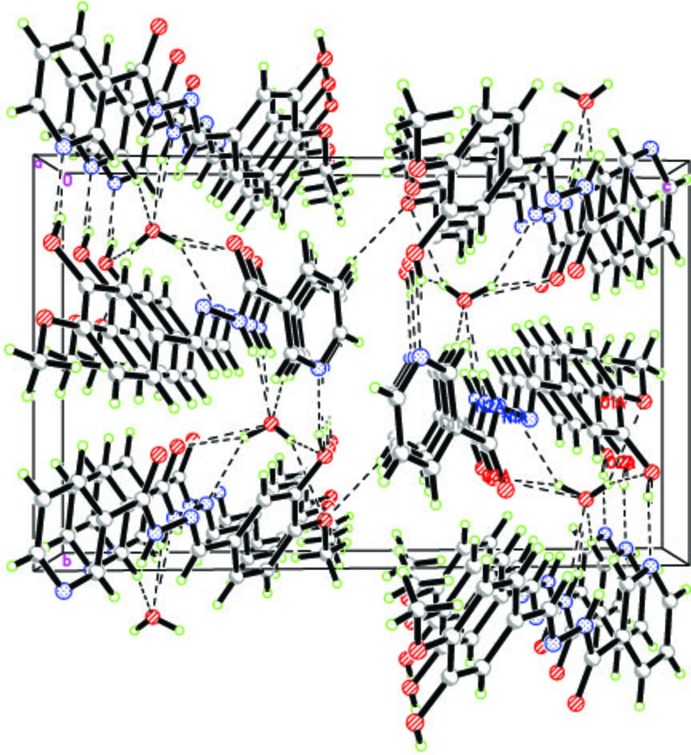
Crystal packing of the title compound, viewed down the *a* axis. Hydrogen bonds are shown as dashed lines.

In the crystal of the title compound (Fig. 2 ▸), the water mol­ecule inter­acts with three neighbouring nicotinohydrazide mol­ecules with the O4 water oxygen atom acting as a hydrogen acceptor through N2—H2*N*⋯O4 and C2—H2⋯O4 hydrogen bonds (Table 1 ▸), and both water H atoms acting as bifurcated donors to form rings of 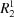
(5) graph-set motif. The nicotinohydrazide mol­ecules are further linked by O—H⋯N and C—H⋯O hydrogen bonds to form a three-dimensional network.

## Hirshfeld surface analysis   

**Figure 3:**
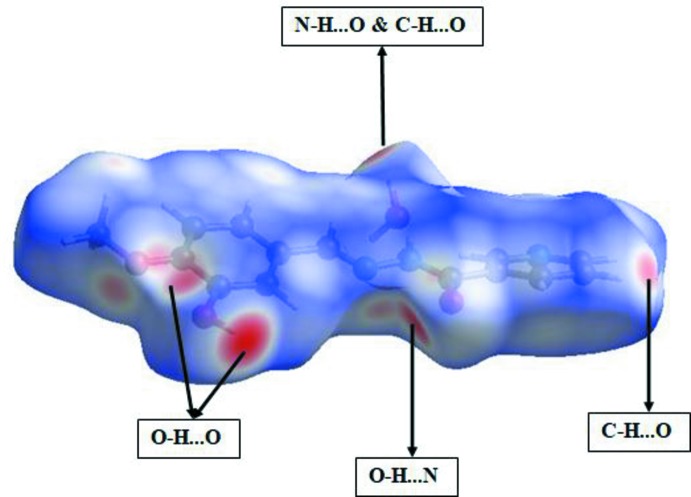
Hirshfeld surfaces of the title compound mapped over *d*_norm_.

**Figure 4:**
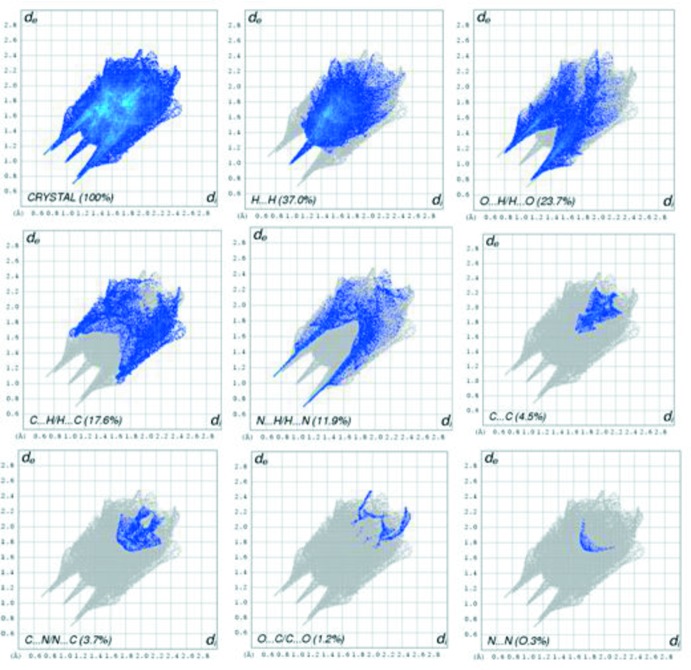
Two-dimensional fingerprint plots for the title compound and relative contributions of the atom pairs to the Hirshfeld surface.

The three-dimensional *d*_norm_ surface is a useful tool for analysing and visualizing the inter­molecular inter­actions, as it shows negative or positive values depending on whether an inter­molecular contact is shorter or longer, respectively, than the sum of the van der Waals radii (Spackman & Jayatilaka, 2009 ▸; McKinnon *et al.*, 2007 ▸). The *d*_norm_ surface of the title compound is shown in Fig. 3 ▸. The red points, which represent closer contacts and negative *d*_norm_ values, correspond to the N—H⋯O, O—H⋯O, O—H⋯N and C—H⋯O inter­actions. Two-dimensional fingerprint plots from the Hirshfeld surface analysis (Fig. 4 ▸) provide information about the inter­molecular contacts and their percentage distributions on the Hirshfeld surface. The percentage of H⋯H contacts as closest contacts on the Hirshfeld surfaces is a universally applicable measure of the crystal lattice energy and can be used as a reference for the importance of other types of contacts. In the title compound, the percentage contributions of the various inter­molecular contacts to the total Hirshfeld surface are as follows: H⋯H (37.0%), C⋯H/H⋯C (17.6%), N⋯H/H⋯N (11.9%), C⋯N/N⋯C (3.7%), O⋯H/H⋯O (23.7%), C⋯C (4.5%), N⋯N (0.3%) and O⋯C/C⋯O (1.2%).

## Frontier mol­ecular orbitals   

**Figure 5:**
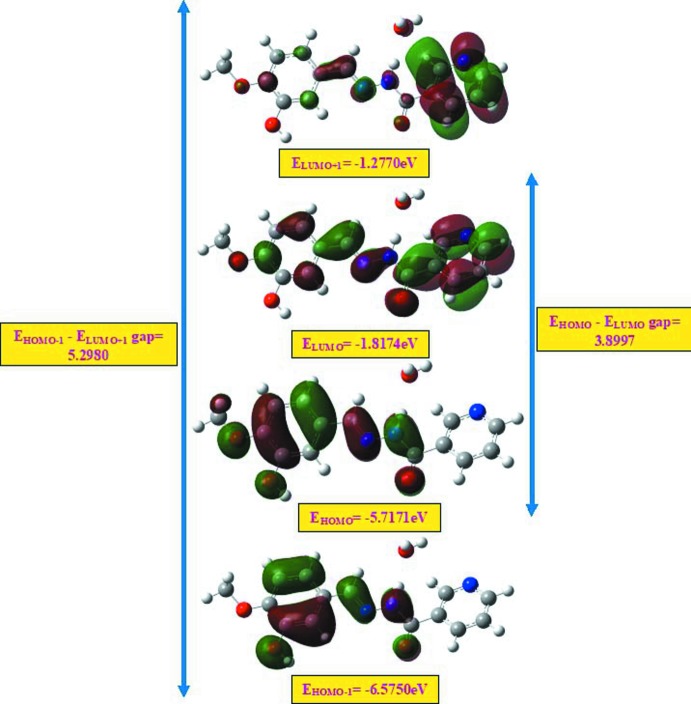
Mol­ecular orbital energy levels of the title compound.

The HOMO (highest occupied mol­ecular orbital) acts as an electron donor and LUMO (lowest occupied mol­ecular orbital) acts as an electron acceptor. If the HOMO–LUMO energy gap is small, then the mol­ecule is highly polarizable and has high chemical reactivity. The energy levels for the title compound were computed by DFT-B3LYP/6-311G++(d,p) method (Sivajeyanthi *et al.*, 2017 ▸). The energy levels, energy gaps, chemical hardness, chemical potential, electronegativity and electrophilicity index are given in Table 2 ▸. As shown in Fig. 5 ▸, the frontier mol­ecular orbital LUMO is located over the whole of the mol­ecule. The energy gap of the mol­ecule clearly shows the charge-transfer inter­action involving donor and acceptor groups. If the HOMO–LUMO energy gap is small, then the mol­ecule is defined as soft, *i.e*. it is highly polarizable and has high chemical reactivity, whereas if the energy gap is large the mol­ecule can be defined as hard. Therefore from Table 2 ▸ we conclude that the title mol­ecule belongs to the really hard materials.

## Database survey   

A search of the Cambridge Structural Database (Version 5.40, update November 2018; Groom *et al.*, 2016 ▸) for uncoordinated *N*′-(benzyl­idene)nicotinohydrazide derivatives O-substituted at the 3,4 positions of the benzene ring yielded three hits, namely *N*′-(1,3-benzodioxol-5-yl­methyl­ene)nicotinohydrazide monohydrate (refcode BUDNIY; Bao *et al.*, 2009 ▸), *N*′-(3,4-di­meth­oxy­benzyl­idene)nicotinohydrazide monohydrate (XODZOH; Novina *et al.*, 2014 ▸) and the isomer *N*′-(4-hy­droxy-3-meth­oxy­benzyl­idene)nicotinohydrazide monohydrate (SEZREV; Shi *et al.*, 2007 ▸). The conformation of the last mol­ecule differs from the title compound mainly in the relative orientation of the pyridine ring with respect to the carbonyl group, as indicated by the value of 158.03 (15)° for the O1—C6—C1—C2 torsion angle in the title compound and of 10.2 (3)° for the corresponding angle in SEZREV. Moreover, in SEZREV the water mol­ecule acts as acceptor of three H atoms from the same nicotinohydrazide mol­ecule and as donor in two O—H⋯O hydrogen bonds.

## Synthesis and crystallization   

The title compound was synthesized by the reaction of a 1:1 molar ratio mixture of a hot ethano­lic solution (20 ml) of nicotinohydrazide (0.137 mg) and a hot ethano­lic solution of 3-hy­droxy-4-meth­oxy benzaldehyde (0.152 mg). After refluxing for 8 h, the solution was then cooled and kept at room temperature to precipitate. Colourless block-shaped crystals suitable for X-ray analysis were obtained by slow evaporation of a 10 ml dimethyl sulfoxide/water (1:1 *v*/*v*) solution.

## Refinement   

Crystal data, data collection and structure refinement details are summarized in Table 3 ▸. H atoms were positioned geom­etrically (O—H = 0.82 Å, N–H = 0.86 Å, C—H = 0.93–0.96 Å) and refined as riding with *U*_iso_(H) = 1.2*U*_eq_(C,N) or 1.5*U*_eq_(O, C-meth­yl)

## Supplementary Material

Crystal structure: contains datablock(s) global, I, 1. DOI: 10.1107/S2056989019006492/rz5252sup1.cif

Structure factors: contains datablock(s) I. DOI: 10.1107/S2056989019006492/rz5252Isup2.hkl

Supporting information file. DOI: 10.1107/S2056989019006492/rz5252Isup3.cml

CCDC reference: 1587259

Additional supporting information:  crystallographic information; 3D view; checkCIF report

## Figures and Tables

**Table 1:** Hydrogen-bond geometry (Å, °)

*D*—H⋯*A*	*D*—H	H⋯*A*	*D*⋯*A*	*D*—H⋯*A*
O4—H4*WA*⋯O2^i^	0.85	2.28	3.0483 (17)	150
O4—H4*WA*⋯O3^i^	0.85	2.49	3.2011 (16)	141
O4—H4*WB*⋯O1^ii^	0.85	2.08	2.8429 (19)	150
O4—H4*WB*⋯N3^ii^	0.85	2.50	3.1875 (18)	139
N2—H2*N*⋯O4	0.86	2.06	2.8889 (18)	162
O2—H10⋯N1^iii^	0.82	1.96	2.7411 (17)	159
C2—H2⋯O4	0.93	2.25	3.129 (2)	156
C4—H4⋯O3^iv^	0.93	2.45	3.347 (2)	163

Symmetry codes: (i) 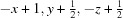; (ii) 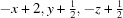; (iii) 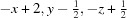; (iv) 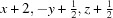.

**Table 2:** Calculated frontier mol­ecular orbital energies (eV)

FMO	Energy
*E*_HOMO_	−5.7171
*E*_LUMO_	−1.8174
*E*_HOMO−1_	−6.5750
*E*_LUMO+1_	−1.2770
*(E*_HOMO_ − *E*_LUMO_) gap	3.8997
*(E*_HOMO−1_ − *E*_LUMO+1_) gap	5.2980
Chemical hardness	1.9498
Chemical potential	3.7672
Electronegativity	−3.7672
Electrophilicity index	3.6393

**Table 3:** Experimental details

Crystal data	
Chemical formula	C_14_H_13_N_3_O_3_·H_2_O
*M*_r_	289.29
Crystal system, space group	Monoclinic, *P*2_1_/*c*
Temperature (K)	295
*a*, *b*, *c* (Å)	7.1153 (4), 11.0075 (6), 18.2771 (10)
β (°)	105.766 (5)
*V* (Å^3^)	1377.64 (14)
*Z*	4
Radiation type	Mo *K*α
μ (mm^−1^)	0.10
Crystal size (mm)	0.30 × 0.25 × 0.18

Data collection	
Diffractometer	Agilent Xcalibur Eos
Absorption correction	Multi-scan (*CrysAlis PRO*; Agilent, 2012 ▸)
*T*_min_, *T*_max_	0.969, 0.981
No. of measured, independent and observed [*I* > 2σ(*I*)] reflections	8396, 2549, 2027
*R*_int_	0.027
(sin θ/λ)_max_ (Å^−1^)	0.606

Refinement	
*R*[*F* ^2^ > 2σ(*F* ^2^)], *wR*(*F* ^2^), *S*	0.036, 0.101, 1.04
No. of reflections	2549
No. of parameters	192
H-atom treatment	H-atom parameters constrained
Δρ_max_, Δρ_min_ (e Å^−3^)	0.16, −0.13

Computer programs: *CrysAlis PRO* (Agilent, 2012 ▸), *SHELXS97* (Sheldrick, 2008 ▸), *SHELXL2017* (Sheldrick, 2015 ▸), *ORTEP-3 for Windows* (Farrugia, 2012 ▸) and *Mercury* (Macrae *et al.*, 2006 ▸).

## supplementary crystallographic information

### Crystal data

**Table:**

C_14_H_13_N_3_O_3_·H_2_O	*F*(000) = 608
*M**_r_* = 289.29	*D*_x_ = 1.395 Mg m^−^^3^
Monoclinic, *P*2_1_/*c*	Mo *K*α radiation, λ = 0.71073 Å
*a* = 7.1153 (4) Å	Cell parameters from 3729 reflections
*b* = 11.0075 (6) Å	θ = 3.9–29.2°
*c* = 18.2771 (10) Å	µ = 0.10 mm^−^^1^
β = 105.766 (5)°	*T* = 295 K
*V* = 1377.64 (14) Å^3^	Block, colourless
*Z* = 4	0.30 × 0.25 × 0.18 mm

### Data collection

**Table:**

Agilent Xcalibur Eos diffractometer	2549 independent reflections
Radiation source: fine-focus sealed tube	2027 reflections with *I* > 2σ(*I*)
Detector resolution: 15.9821 pixels mm^-1^	*R*_int_ = 0.027
ω scans	θ_max_ = 25.5°, θ_min_ = 3.9°
Absorption correction: multi-scan (CrysAlis PRO; Agilent, 2012)	*h* = −8→8
*T*_min_ = 0.969, *T*_max_ = 0.981	*k* = −13→12
8396 measured reflections	*l* = −22→22

### Refinement

**Table:**

Refinement on *F*^2^	Secondary atom site location: difference Fourier map
Least-squares matrix: full	Hydrogen site location: mixed
*R*[*F*^2^ > 2σ(*F*^2^)] = 0.036	H-atom parameters constrained
*wR*(*F*^2^) = 0.101	*w* = 1/[σ^2^(*F*_o_^2^) + (0.0462*P*)^2^ + 0.2987*P*] where *P* = (*F*_o_^2^ + 2*F*_c_^2^)/3
*S* = 1.04	(Δ/σ)_max_ < 0.001
2549 reflections	Δρ_max_ = 0.16 e Å^−^^3^
192 parameters	Δρ_min_ = −0.13 e Å^−^^3^
0 restraints	Extinction correction: SHELXL2017 (Sheldrick, 2015), Fc^*^=kFc[1+0.001xFc^2^λ^3^/sin(2θ)]^-1/4^
Primary atom site location: structure-invariant direct methods	Extinction coefficient: 0.030 (3)

### Special details

**Table:**

Geometry. All esds (except the esd in the dihedral angle between two l.s. planes) are estimated using the full covariance matrix. The cell esds are taken into account individually in the estimation of esds in distances, angles and torsion angles; correlations between esds in cell parameters are only used when they are defined by crystal symmetry. An approximate (isotropic) treatment of cell esds is used for estimating esds involving l.s. planes.
Refinement. Refinement of F^2^ against ALL reflections. The weighted R-factor wR and goodness of fit S are based on F^2^, conventional R-factors R are based on F, with F set to zero for negative F^2^. The threshold expression of F^2^ > 2sigma(F^2^) is used only for calculating R-factors(gt) etc. and is not relevant to the choice of reflections for refinement. R-factors based on F^2^ are statistically about twice as large as those based on F, and R- factors based on ALL data will be even larger.

### Fractional atomic coordinates and isotropic or equivalent isotropic displacement parameters (Å^2^)

**Table:**

	*x*	*y*	*z*	*U*_iso_*/*U*_eq_	
O3	−0.03769 (14)	0.40424 (10)	0.06695 (6)	0.0455 (3)	
O2	0.20750 (15)	0.23383 (10)	0.06507 (7)	0.0537 (3)	
H10	0.287088	0.178188	0.073527	0.081*	
O1	1.15498 (17)	0.21431 (11)	0.30304 (8)	0.0604 (4)	
N3	0.85559 (17)	0.37007 (12)	0.26300 (7)	0.0418 (3)	
N2	1.03456 (17)	0.40123 (12)	0.31206 (7)	0.0410 (3)	
H2N	1.053128	0.472235	0.332397	0.049*	
N1	1.61018 (19)	0.51282 (12)	0.42610 (8)	0.0452 (4)	
C8	0.5291 (2)	0.43898 (15)	0.20366 (8)	0.0371 (4)	
C9	0.4682 (2)	0.33704 (14)	0.15750 (8)	0.0384 (4)	
H9	0.555924	0.274429	0.157443	0.046*	
C10	0.2791 (2)	0.32888 (14)	0.11216 (8)	0.0372 (4)	
C11	0.1465 (2)	0.42290 (14)	0.11294 (8)	0.0355 (4)	
C12	0.2059 (2)	0.52410 (15)	0.15746 (9)	0.0409 (4)	
H12	0.118419	0.586791	0.157676	0.049*	
C13	0.3972 (2)	0.53171 (15)	0.20196 (9)	0.0423 (4)	
H13	0.437730	0.600772	0.231319	0.051*	
C14	−0.1795 (2)	0.49637 (17)	0.06553 (10)	0.0492 (4)	
H14A	−0.192906	0.508196	0.115902	0.074*	
H14B	−0.302791	0.472017	0.032256	0.074*	
H14C	−0.138193	0.570937	0.047468	0.074*	
C7	0.7260 (2)	0.45278 (15)	0.25379 (8)	0.0411 (4)	
H7	0.758441	0.525352	0.280265	0.049*	
C6	1.1785 (2)	0.31829 (14)	0.32729 (9)	0.0390 (4)	
C2	1.4323 (2)	0.47808 (14)	0.38578 (8)	0.0387 (4)	
H2	1.343594	0.537888	0.362746	0.046*	
C1	1.3728 (2)	0.35815 (13)	0.37638 (8)	0.0354 (4)	
C5	1.5059 (2)	0.27035 (15)	0.41019 (10)	0.0502 (4)	
H5	1.472088	0.188603	0.404400	0.060*	
C4	1.6891 (2)	0.30435 (17)	0.45260 (11)	0.0599 (5)	
H4	1.780614	0.246398	0.476287	0.072*	
C3	1.7334 (2)	0.42511 (17)	0.45907 (10)	0.0533 (5)	
H3	1.857046	0.447633	0.488254	0.064*	
O4	1.07802 (17)	0.65762 (11)	0.34578 (7)	0.0594 (4)	
H4WA	1.034030	0.696974	0.377588	0.089*	
H4WB	1.043530	0.692674	0.302788	0.089*	

### Atomic displacement parameters (Å^2^)

**Table:**

	*U*^11^	*U*^22^	*U*^33^	*U*^12^	*U*^13^	*U*^23^
O3	0.0286 (5)	0.0448 (7)	0.0558 (7)	0.0044 (5)	−0.0009 (5)	−0.0023 (5)
O2	0.0385 (6)	0.0378 (6)	0.0712 (8)	0.0062 (5)	−0.0085 (5)	−0.0115 (6)
O1	0.0513 (7)	0.0382 (7)	0.0744 (9)	−0.0022 (5)	−0.0127 (6)	−0.0079 (6)
N3	0.0309 (6)	0.0455 (8)	0.0416 (7)	−0.0061 (6)	−0.0029 (5)	0.0026 (6)
N2	0.0311 (6)	0.0390 (7)	0.0448 (7)	−0.0032 (6)	−0.0037 (5)	−0.0026 (6)
N1	0.0361 (7)	0.0419 (8)	0.0509 (8)	−0.0062 (6)	0.0006 (6)	0.0030 (6)
C8	0.0317 (7)	0.0424 (9)	0.0351 (8)	−0.0033 (7)	0.0054 (6)	0.0051 (7)
C9	0.0306 (7)	0.0373 (8)	0.0437 (8)	0.0036 (6)	0.0039 (6)	0.0062 (7)
C10	0.0343 (8)	0.0327 (8)	0.0408 (8)	−0.0019 (6)	0.0038 (6)	0.0024 (7)
C11	0.0281 (7)	0.0388 (9)	0.0369 (8)	−0.0003 (6)	0.0039 (6)	0.0052 (7)
C12	0.0369 (8)	0.0403 (9)	0.0435 (8)	0.0051 (7)	0.0073 (6)	−0.0004 (7)
C13	0.0399 (8)	0.0422 (9)	0.0412 (8)	−0.0020 (7)	0.0049 (7)	−0.0052 (7)
C14	0.0325 (8)	0.0598 (11)	0.0520 (10)	0.0118 (8)	0.0056 (7)	0.0013 (8)
C7	0.0348 (8)	0.0446 (9)	0.0397 (8)	−0.0055 (7)	0.0030 (6)	0.0012 (7)
C6	0.0370 (8)	0.0345 (9)	0.0398 (8)	−0.0047 (7)	0.0007 (6)	0.0025 (7)
C2	0.0320 (8)	0.0364 (8)	0.0432 (8)	−0.0003 (6)	0.0023 (6)	0.0041 (7)
C1	0.0321 (7)	0.0359 (8)	0.0345 (7)	−0.0008 (6)	0.0026 (6)	0.0024 (6)
C5	0.0457 (9)	0.0361 (9)	0.0580 (10)	−0.0003 (7)	−0.0041 (8)	0.0050 (8)
C4	0.0432 (10)	0.0477 (11)	0.0728 (13)	0.0060 (8)	−0.0114 (9)	0.0120 (9)
C3	0.0328 (8)	0.0540 (11)	0.0611 (11)	−0.0043 (8)	−0.0075 (7)	0.0063 (9)
O4	0.0627 (8)	0.0475 (7)	0.0583 (7)	0.0186 (6)	−0.0002 (6)	−0.0017 (6)

### Geometric parameters (Å, º)

**Table:**

O3—C11	1.3664 (17)	C12—C13	1.386 (2)
O3—C14	1.4257 (19)	C12—H12	0.9300
O2—C10	1.3627 (18)	C13—H13	0.9300
O2—H10	0.8198	C14—H14A	0.9600
O1—C6	1.2223 (19)	C14—H14B	0.9600
N3—C7	1.274 (2)	C14—H14C	0.9600
N3—N2	1.3866 (16)	C7—H7	0.9300
N2—C6	1.343 (2)	C6—C1	1.4950 (19)
N2—H2N	0.8602	C2—C1	1.383 (2)
N1—C3	1.333 (2)	C2—H2	0.9300
N1—C2	1.3355 (19)	C1—C5	1.376 (2)
C8—C13	1.381 (2)	C5—C4	1.376 (2)
C8—C9	1.400 (2)	C5—H5	0.9300
C8—C7	1.459 (2)	C4—C3	1.364 (3)
C9—C10	1.378 (2)	C4—H4	0.9300
C9—H9	0.9300	C3—H3	0.9300
C10—C11	1.404 (2)	O4—H4WA	0.8500
C11—C12	1.377 (2)	O4—H4WB	0.8495
			
C11—O3—C14	117.37 (12)	H14A—C14—H14B	109.5
C10—O2—H10	109.5	O3—C14—H14C	109.5
C7—N3—N2	114.41 (13)	H14A—C14—H14C	109.5
C6—N2—N3	118.71 (13)	H14B—C14—H14C	109.5
C6—N2—H2N	120.6	N3—C7—C8	123.07 (15)
N3—N2—H2N	120.7	N3—C7—H7	118.5
C3—N1—C2	116.76 (14)	C8—C7—H7	118.5
C13—C8—C9	118.80 (13)	O1—C6—N2	122.66 (13)
C13—C8—C7	117.90 (14)	O1—C6—C1	120.37 (14)
C9—C8—C7	123.31 (14)	N2—C6—C1	116.97 (13)
C10—C9—C8	120.43 (14)	N1—C2—C1	123.65 (14)
C10—C9—H9	119.8	N1—C2—H2	118.2
C8—C9—H9	119.8	C1—C2—H2	118.2
O2—C10—C9	124.50 (13)	C5—C1—C2	117.67 (13)
O2—C10—C11	115.81 (12)	C5—C1—C6	118.33 (14)
C9—C10—C11	119.69 (14)	C2—C1—C6	123.84 (13)
O3—C11—C12	125.07 (13)	C4—C5—C1	119.55 (15)
O3—C11—C10	114.71 (13)	C4—C5—H5	120.2
C12—C11—C10	120.22 (13)	C1—C5—H5	120.2
C11—C12—C13	119.40 (14)	C3—C4—C5	118.38 (15)
C11—C12—H12	120.3	C3—C4—H4	120.8
C13—C12—H12	120.3	C5—C4—H4	120.8
C8—C13—C12	121.44 (15)	N1—C3—C4	123.96 (15)
C8—C13—H13	119.3	N1—C3—H3	118.0
C12—C13—H13	119.3	C4—C3—H3	118.0
O3—C14—H14A	109.5	H4WA—O4—H4WB	109.5
O3—C14—H14B	109.5		

### Hydrogen-bond geometry (Å, º)

**Table:**

*D*—H···*A*	*D*—H	H···*A*	*D*···*A*	*D*—H···*A*
O4—H4*WA*···O2^i^	0.85	2.28	3.0483 (17)	150
O4—H4*WA*···O3^i^	0.85	2.49	3.2011 (16)	141
O4—H4*WB*···O1^ii^	0.85	2.08	2.8429 (19)	150
O4—H4*WB*···N3^ii^	0.85	2.50	3.1875 (18)	139
N2—H2*N*···O4	0.86	2.06	2.8889 (18)	162
O2—H10···N1^iii^	0.82	1.96	2.7411 (17)	159
C2—H2···O4	0.93	2.25	3.129 (2)	156
C4—H4···O3^iv^	0.93	2.45	3.347 (2)	163

Symmetry codes: (i) −*x*+1, *y*+1/2, −*z*+1/2; (ii) −*x*+2, *y*+1/2, −*z*+1/2; (iii) −*x*+2, *y*−1/2, −*z*+1/2; (iv) *x*+2, −*y*+1/2, *z*+1/2.
